# Influence of graft size, histocompatibility,and cryopreservation on reproductive outcome following ovary transplantation in mice

**DOI:** 10.1007/s10815-019-01620-9

**Published:** 2019-11-18

**Authors:** T. Kolbe, I. Walter, T. Rülicke

**Affiliations:** 1 Biomodels Austria, University of Veterinary Medicine Vienna, Vienna, Austria; 2 Department IFA Tulln, University of Natural Resources and Life Sciences, Tulln, Austria; 3 Vetcore (VetBioBank), University of Veterinary Medicine Vienna, Vienna, Austria; 4 Institute of Laboratory Animal Science, University of Veterinary Medicine Vienna, Vienna, Austria

**Keywords:** ovary transplantation, mouse, cryopreservation, graft size, immunodeficiency

## Abstract

**Purpose:**

Transplantation of ovarian tissue is a valuable method to rescue mouse strains with fertility problems and to revitalize archived strains. The purpose of this study was to investigate the effect of (i) different sizes of transplanted ovary pieces on reproductive outcome, (ii) use of immunodeficient recipients to overcome the limitation of histocompatibility, and (iii) to compare different protocols for cryopreservation of ovarian tissue.

**Methods:**

Halves, quarters, and eights of mouse ovaries were transplanted. Half ovaries from B6 donors were transferred into immunodeficient mice. Halves of ovaries were frozen according to four different protocols, thawed and transferred.

**Results:**

Pregnancy rate after transplantation of ovarian tissue was high (90–100%) independent of the transplant size. Although, the average litter size was significantly lower for recipients of quarters and eights (4.4 and 4.6 vs. 6.5), the total number of offspring produced per donor ovary was higher compared with recipients of halves. Pregnancy rate of immunodeficient recipients was 40% (mean 4.7 offspring per litter). All four cryopreservation protocols used were able to preserve functionality of the ovarian tissue.

**Conclusions:**

Transplantation of ovarian tissue smaller than halves resulted in reduced litter sizes. The distribution of ovarian tissue of one donor female to 4 or 8 recipients will therefore yield in a higher total number of offspring in a certain time period. The use of immunodeficient recipients is an option for non-histocompatible donors. Cryopreservation of ovarian tissue is generally feasible but the function of frozen-thawed ovary halves after transplantation differs depending on the freezing protocol used.

## Introduction

Ovary transfer in mice has been used since the last century for experimental reasons [1]. Meanwhile, the transplantation of ovarian tissue is applied to save valuable mouse lines with fertility problems and to revitalize cryopreserved mouse strains archived as ovarian tissue in mouse repositories [2–8]. Moreover, the breeding of genetically modified lines with serious welfare problems can be refined by transplantation of their ovaries into healthy wildtype recipients. For successful reconstitution of recipient fertility, ovaries have to be either freshly transferred or archived within 2 h without cooling [9] or within 4 h stored at 4 °C [10] after death of the donor and transferred after thawing. Mostly whole ovaries are transferred [1, 2, 6–8, 11–13], but in some studies, half ovaries are used [3–5, 8, 9] and sometimes even quarter pieces [9, 14]. To split the ovaries into smaller pieces simplified the transfer procedure compared with the transfer of whole ovaries.

Additionally, in cryopreservation studies, the permeation of tissue with cryoprotectants was improved by reduction of the tissue size.

However, published results between different studies are not comparable because of the use of different parameters like donor age (from day 16 of pregnancy [2] via 10–16 days [6, 7, 13] up to 12 weeks [11]), mouse strains (BALB/c [2, 7], FVB [4], C57BL/6 [13], C3H [8], ICR [9], or DBA [1]), recipient age (4 weeks [9, 13] up to 12 weeks [11], penetrating cryoprotective agent (DMSO [2–4, 6–8, 11, 12, 14], ethylene glycol [7], DMSO + ethylene glycol [14], DMSO + acetamide + propylene glycol [13], DMSO + acetamide + propylene glycol + polyethylene glycol [9]), or the cryopreservation method (vitrification [9, 13, 14] or slow freezing [2–9, 11, 12, 14]). To our knowledge, there is no systematic comparison of the transfer of different sized ovary pieces in mice.

A prerequisite for engraftment of transplanted tissue is the histocompatibility between donor and recipient determined by the genes of the major histocompatibility complex (MHC). Without histocompatibility, the transplant will be rejected. Therefore, donors of outbred strains with undefined mixed genetic background are not suitable because of the absence of appropriate histocompatible recipients. In the International Mouse Strain Resource (IMSR), more than 286,000 strains are listed but only 883 are indicated as inbred or major histocompatible (www.findmice.org). A possible strategy to overcome this incompatibility problem is to use very young recipients without a fully developed immune system [9]. But this approach induces technical problems due to the small size of the very young recipients. Alternatively, adult immunodeficient mouse mutants could serve as universal recipients but are still rarely used for the transplantation of mouse ovaries [12, 14]. In contrast, immunodeficient mice have frequently been used for xenotransplantation as recipients for ovary pieces from the rat [15], human [16–21], sheep [22, 23], cat [24, 25], cattle [26, 27], wombat [28], elephant [29], dog [30, 31], and lion [32].

Cryopreservation of ovarian tissue offers a valuable additional resource to the routinely used gametes and embryos for archiving and fertility recovery. In reproductive medicine, freezing of ovaries with subsequent thawing and surgical engraftment can restore fertility in young women undergoing cancer treatment with chemo- and/or radiotherapy [33–36]. Moreover, ovarian tissue can be a valuable tool to preserve important lines or strains of laboratory, domestic, and wild animals. Frozen ovaries may offer a valuable addition to the current cryopreservation approaches with embryos and gametes. Although different protocols for the cryopreservation of mouse ovaries were published, a direct comparison of fertility parameters after transplantation of thawed ovarian tissue is still missing [2–7, 11, 12, 37–39]. We have selected four very different protocols from the literature with regards to the physical process (vitrification, slow freezing), containment (straw, cryo tube), and cryoprotective agent (dimethyl sulfoxide (DMSO), acetamide, ethylene glycol).

Here, we address different aspects of transplantation and cryopreservation of ovarian tissue of mice in order to maximize the benefit of the procedures for practical application.

## Materials and methods

### Animals and husbandry conditions

All animals were housed in Macrolon® cages, lined with bedding (Lignocel®, heat treated, Rettenmaier Austria Gmbh & Co. KG, Vienna, Austria), and enriched with nesting material (Pur-Zellin 4 × 5 cm; Paul Hartmann GesmbH, Wiener Neudorf, Austria), in a rodent facility (room temperature 21 ± 1 °C [mean ± SEM]; relative humidity 40–55%; photoperiod 12L:12D). Food (V1126, Ssniff Spezialdiaeten GmbH, Soest, Germany) and tap water were available ad libitum. The specific pathogen-free status was confirmed quarterly according to FELASA (Federation for Laboratory Animal Science Associations) recommendations [40]. Experimental procedures were discussed and approved by the institutional ethics committee and granted by the national authority under license numbers BMWF-68.205/0258-II/3b/2011, BMWF-68.205/0023-II/3b/2014 and BMBWF-68.205/0062-V/3b/2018.

### Surgical transfer

Ovary donors were sacrificed by cervical dislocation immediately before transplantation and dissected ovaries were stored in M2 medium (M7167-100 ml, Sigma-Aldrich Handels GmbH, Vienna, Austria) at 37 °C for at most 60 min. Recipients were anesthetized by intraperitoneal (i.p.) injection of Ketamine/Xylazine (100 mg/kg BW Ketamine (Ketasol), 4 mg/kg BW Xylazine (Rompun), Graeub Veterinary Products, Bern, Switzerland) and eyes were covered with eye ointment (Oleovit, Fresenius Kabi, Graz, Austria) during surgery. After disinfection with 70% ethanol, an incision of the skin and the peritoneum was made lateral close to the ovary for gentle extraction of the ipsilateral reproductive tract. To unwrap the ovary, the ovarian bursa, a transparent tissue membrane that covers the ovary, was opened with fine iris scissors for a distance of a few millimeters directly along the ovarian fat pad. For ovariectomy, the ovarian ligament which held the ovary in place was cut with fine scissors. After stopping bleeding from the cut vessels, a new piece of ovarian tissue (according to the experimental protocol: experiment 1, 2, or 3) was orthotopically grafted without fixation and wrapped with the bursa. Then, the reproductive tract was gently placed back into the abdominal cavity, the peritoneum was sutured, and the skin was closed with Michel clamps. The whole procedure was repeated on the contralateral side. Post operation analgesia was applied by subcutaneous (s.c.) injection of 5 mg/kg BW Meloxicam (Metacam, Boehringer Ingelheim Vetmedica GmbH, Ingelheim am Rhein, Germany). The mice were kept on a warming plate until complete recovery from anesthesia. After a recovery period of 10 days, recipient females were mated with proven males to examine their fertility.

### Experiment 1 (size of the ovary pieces)

**Fig. 1:**
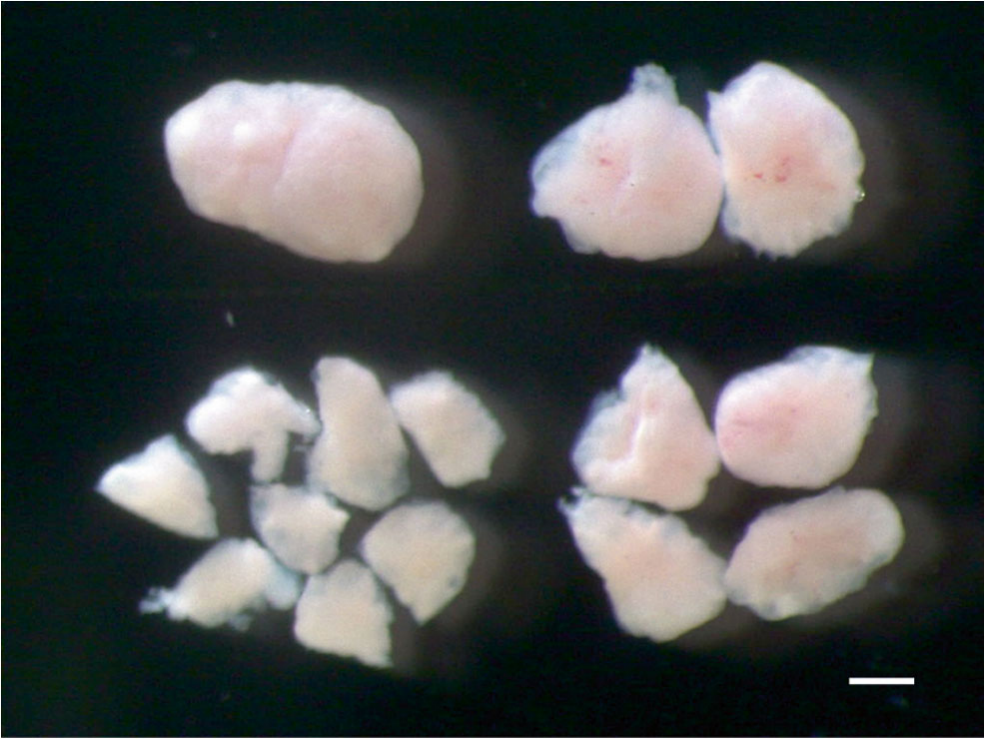
Dissected whole mouse ovary (left upper side), two halves of a mouse ovary (right upper side), four quarters of a mouse ovary (left lower side), and eight eighths of a mouse ovary (right lower side) prepared for transplantation (scale bar = 1 mm)

Fresh dissected ovaries of C57BL/6N origin (donor age 8–12 weeks) were sliced into 2, 4, and 8 equal pieces (Fig. 1). After ovariectomy, three groups of each 10 females of the strain tdTomato (B6.129-Gt(ROSA)26Sor^tm4(ACTB-tdTomato,-EGFP)Luo^/J, The Jackson Laboratory, Bar Harbor, USA, Jax Stock No. 007676) aging 8–10 weeks received bilateral transplants of the same size (halves, quarters, or eighths). Ten days after surgery, Michel clamps were removed and females were permanently mated with C57BL/6N or B6D2F1 males (2–8 months old, equally distributed between groups) for 4 months. Days from mating to birth of the first litter (latency), pregnancy rate, litter size, and number of litters were recorded. To confirm parentage, the presence or absence of red fluorescence of newborn pups was examined using a blue light fluorescence lamp with an excitation maximum of 554 nm, emission maximum of 581 nm was visible with a GFSP-5 headset (BLS Ltd., Budapest, Hungary).

### Experiment 2 (immunodeficient recipients)

Half ovaries of tdTomato donor mice (age 8–14 weeks) were transplanted into 10 immunodeficient NSG recipients (NOD.Cg-*Prkdc*^*scid*^*-Il2rg*^*tm1Wjl*^/SzJ, Jax Stock No. 005557, age 9–14 weeks). After recovery of 10 days, females were permanently mated with NSG males (age 3–5 months) for a period of 4 months. To confirm parentage, the presence or absence of red fluorescence excitation emission of newborn pups was examined. Data collection of born offspring was performed as described for experiment 1.

### Experiment 3 (cryopreservation)

To compare different cryopreservation protocols, two vitrification protocols and two conventional freezing protocols were tested.

Ovaries from 25 C57BL/6N females (age 5 months) were collected and cut into two equal pieces (Fig. 1, right upper corner). All four half ovaries of a donor were frozen together and two half ovaries of the same donor were transplanted to a recipient after thawing. The ovarian tissues from all donors were allocated to the following groups:

#### Group I (protocol according to [13], based on [37])

Freezing: Tissues were pretreated in phosphate buffered medium PBI [41] with 1 M DMSO at room temperature (RT), then transferred with 5 μl PBI into cryo vials at 0 °C for 5 min. Ninety-five-milliliter DAP123 (2 M DMSO, 1 M Acetamid, 3 M propylene glycol) was added, tube stored again at 0 °C for 5 min and plunged directly into liquid nitrogen.

Thawing: 30 sec at RT, diluted with 900 μl PBI with 0.25 M sucrose at 37 °C without agitation.

#### Group II (vitrification protocol according to [14])

Freezing: Tissues were incubated in KSOM-H medium (potassium-supplemented simplex optimized medium (KSOM) with 2-(4-(2-Hydroxyethyl)-1-piperazinyl)-ethansulfonsäure (H: HEPES)) with 1.5 M ethylene glycol and 1.5 M DMSO for 20 min at RT, then transferred into KSOM-H with 2.55 M ethylene glycol, 2.55 M DMSO, and 0.75 M sucrose for 3 min at RT. Tissues were placed on an ultracooled surface (~ 196 °C) [42] and stored in precooled cryo vials.

Thawing: Tissues given in KSOM-H with 0.5 M sucrose at 37 °C for 5 min without agitation and then transferred through decreasing concentrations of sucrose (0.25 M, 0.125 M, 0 M) in 5 min intervals.

#### Group III (slow cooling protocol according to [14], based on [7])

Freezing: Tissues were equilibrated in PBS (phosphate buffered saline) with 1.5 M DMSO and 0.1 M sucrose for 10 min at RT, filled in 0.5 ml straws, sealed and cooled by a rate of 2 °C/min to − 7 °C, seeded, kept for 10 min, and cooled down again by a rate of 0.3 °C/min to − 40 °C, then plunged into liquid nitrogen.

Thawing: Straws held for 10 s in liquid nitrogen vapor, 30 s at RT, and 20 s in a 37 °C water bath. Ends were cut, the content emptied into KSOM-H with 0.5 M sucrose for 5 min at RT without agitation and then transferred through decreasing concentrations of sucrose (0.25 M, 0.125 M, 0 M) in 5 min intervals.

#### Group IV (protocol according [8])

Freezing: Tissues were placed into 300 μl of M2 with 10% FBS (fetal bovine serum, 10270-098, ThermoFisher Scientific, Vienna, Austria) and 1.5 M DMSO in a cryo vial, equilibrated for 10 min at RT, put in a freezer at 0 °C for 20 min, cooled down by a rate of 2 °C/min to − 7 °C, seeded, kept for 10 min, and cooled down again by a rate of 0.3 °C/min to − 40 °C, then plunged into liquid nitrogen.

Thawing: Vials were held for 40 s at RT then were placed into 30–37 °C warm water until thawed. Tissues were transferred into 2 ml of M2 for 5 min at RT without agitation and washed two times in M2.

#### Group V

Control, transplantation of fresh ovary halves.

Frozen samples of each freezing protocol were stored in liquid nitrogen for 1 month until thawing. Transplantation of the thawed ovarian tissues was immediately done after ovariectomy of the C57BL/6N recipients. The control group was transplanted with halves of fresh prepared ovaries of the same inbred strain.

After full recovery, recipients of all fife groups were mated with C57BL/6N males (age 5 months) for a maximum of 100 days. Once pregnancy was visually validated, females were separated from their stud male. Investigation of born offspring and grafts was performed as described for experiment 1.

### Histology

To investigate the functionality of ovary grafts, the recipients were sacrificed at the end of the mating period. After gross examination of the position and general appearance, the grafts were histologically analyzed. Dissected ovary pieces were immediately fixed in 4% buffered formaldehyde solution and subsequently embedded in paraffin. Serial sections were stained with Hematoxylin & Eosin (H&E). Ovaries from two untreated C57BL/6N females were used as controls for tissue integrity and position of follicles between ovarian tissue and connective hilus.

### Statistical procedures

To compare the measured data of the different treatment groups, we used IBM SPSS Statistics V24. Pregnancy rates were compared by Fisher’s exact test. Latency (time from mating until birth of pups) and litter sizes were compared by one-way ANOVA. Litter numbers in experiment 1 were compared by the Kruskal Wallis test. Differences between groups in experiment 3 were analyzed using Tukey’s post hoc procedure. Two-tailed *p* values of *p* ≤ 0.05 were considered to be statistically significant.

## Results

### Experiment 1 (size of the ovary pieces)

**Fig. 2:**
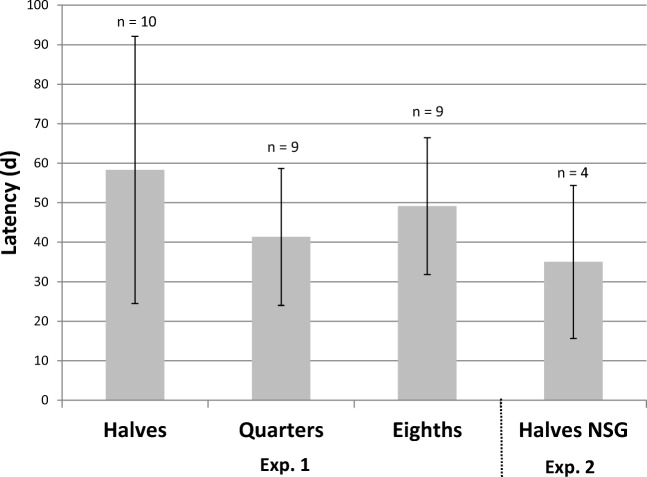
Mean and standard deviation of latency in days after transfer of half, quarter, and eighth ovary pieces into immunocompetent recipients and half ovary pieces into immunodeficient recipients (*n* = number of fertile recipients per group)

**Fig. 3:**
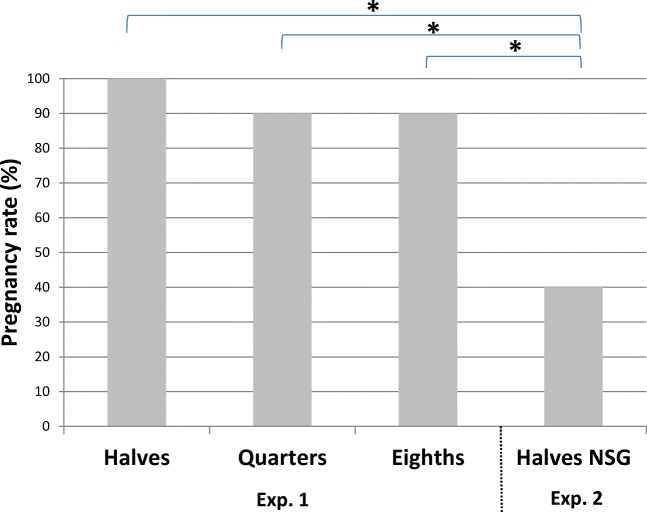
Pregnancy rates after transfer of half, quarter, and eighth ovary pieces into immunocompetent recipients and half ovary pieces into immunodeficient recipients (*n* = 10 per group, *p ≤ 0.05, ANOVA)

**Fig. 4:**
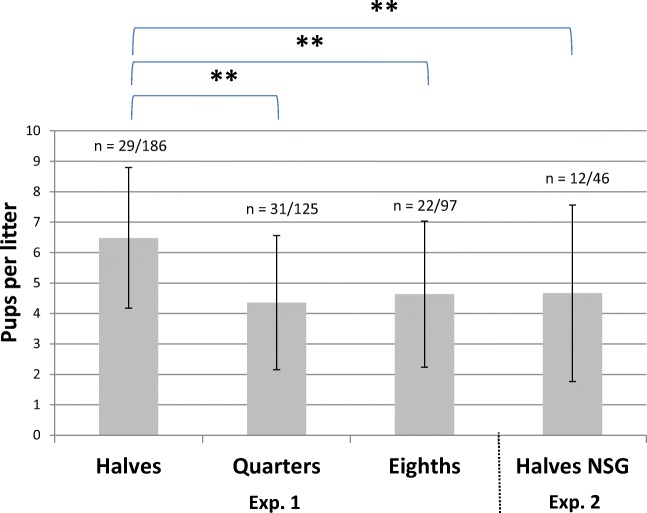
Mean and standard deviation of born pups per litter after transfer of half, quarter, and eighth ovary pieces into immunocompetent recipients and half ovary pieces into immunodeficient recipients (*n* = total number of litters/total number of donor offspring per group, **p ≤ 0.01, ANOVA)

Differences in latency in days between groups (group 1: 58.3, group 2: 41.3, group 3: 49.1) were not significant, probably due to a high variability within groups (Fig. 2). Some recipients gave birth 21 days after mating indicating that the transplant was fully functional after 10 days of recovery. A high pregnancy rate (90–100%) was observed for all groups independent of graft size (Fig. 3). However, litter sizes were significantly lower in transfer groups with quarters and eighths compared with halves (4.4 ± 2.2 and 4.6 ± 2.4 vs. 6.5 ± 2.3, *p* ≤ 0.01, ANOVA, Fig. 4). The average number of litters per fertile recipient during the 4-month observation period was 2.9 for the group with half pieces, 3.4 for quarters, and 2.4 litters, the lowest, for the recipients of eighths (data not shown). The difference was not statistically different due to a high standard deviation in group 1 (1.73 vs. 0.88 and 0.73; *p* > 0.05, Kruskal Wallis test). Transplantation of halves resulted in 29 litters with 186 pups in total, quarters in 31 litters with 125 pups, and eights in 22 litters with 97 pups.

**Fig. 5:**
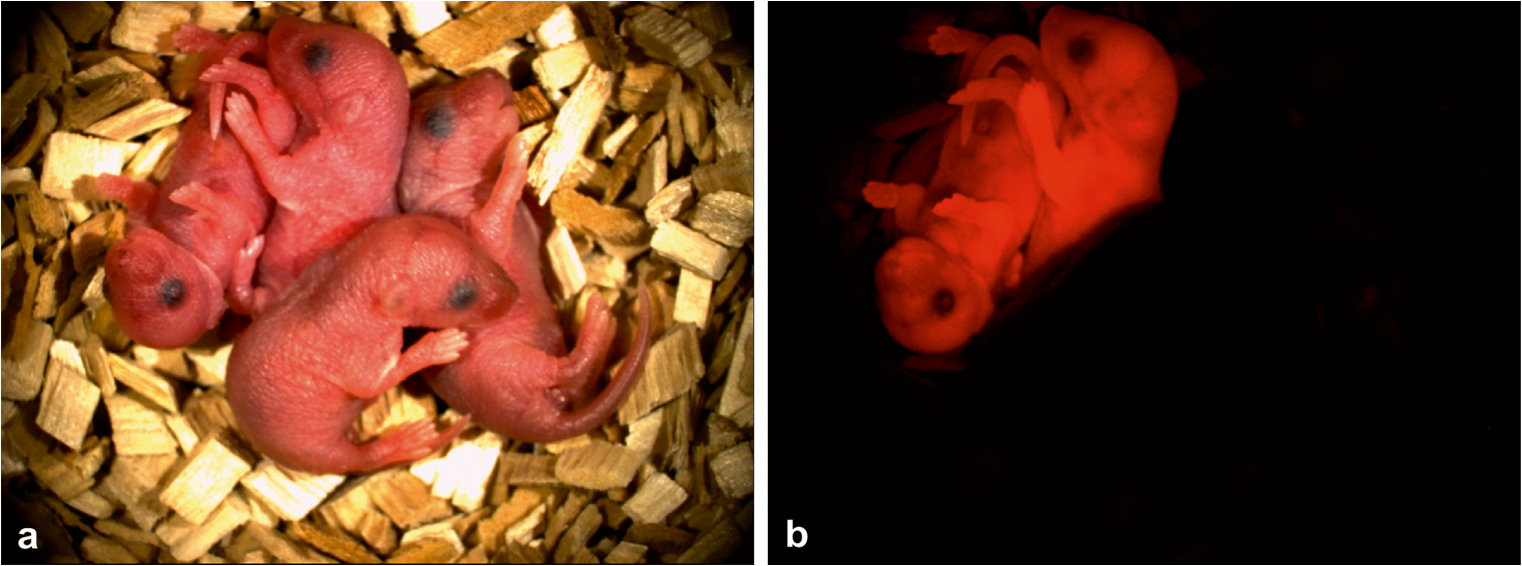
Detection of offspring in a litter of a tdTomato recipient transplanted with wildtype ovarian tissue. Expression of red fluorescence protein of two newborn in a litter demonstrate remaining recipient germ cells. **a** Bright light. **b** Red fluorescence excitation emission

**Fig. 6:**
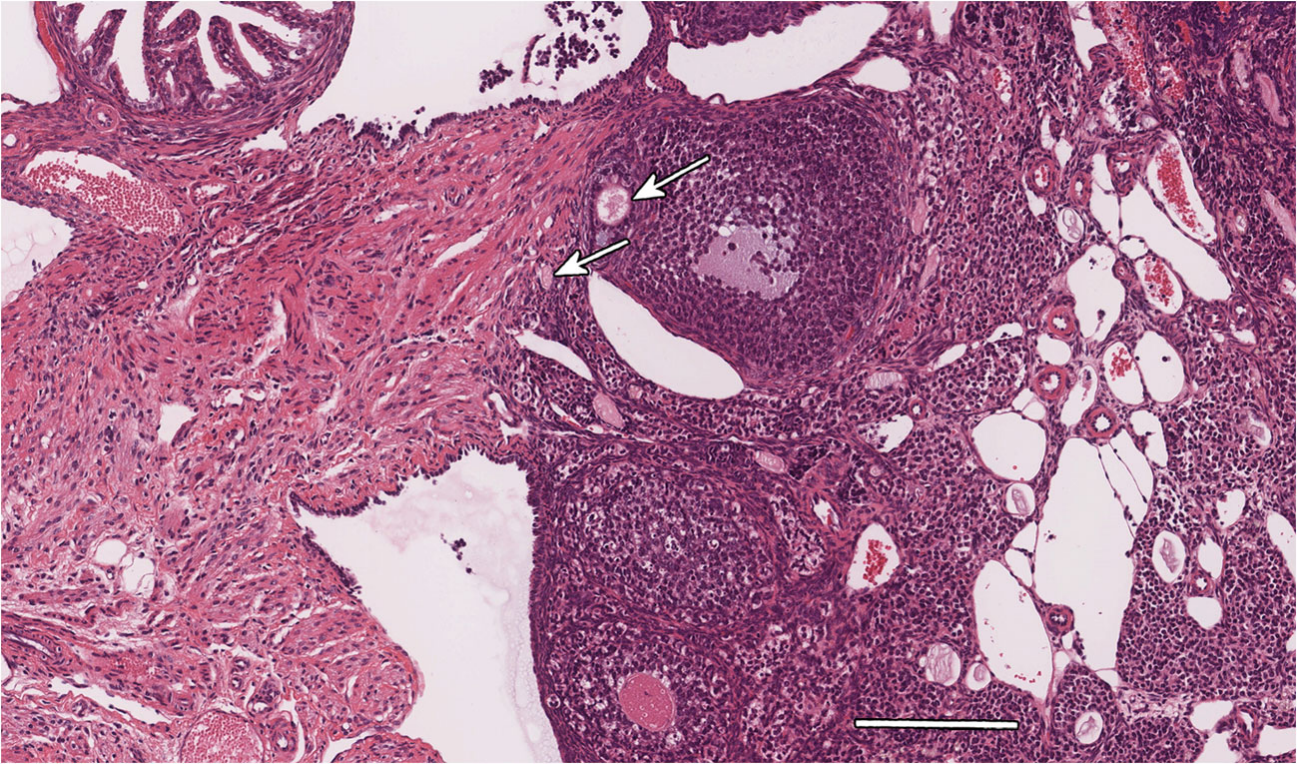
H&E staining of a dissected ovary with hilus of an untreated C57BL/6N mouse. Arrows indicate follicles at the borderline between ovary and hilus (H&E staining, × 100 magnification)

Interestingly, transplantation of ovarian tissue from wt donors to tdTomato recipients revealed that a few pups originated from oocytes of residual ovarian tissue (4.2%, 17/407, Fig. 5). H&E staining of dissected grafts showed that follicles extend to the stem (hilus) of the ovary, which could not be removed completely (Fig. 6). Offspring with tdTomato expression was not considered in the calculation of reproductive success of recipients.

### Experiment 2 (immunodeficient recipients)

The latency in days after transfer was comparable with the other transfer groups (Fig. 2). In contrast to the high pregnancy rates of 90–100% of immunocompetent C57BL/6N recipients in experiment 1, pregnancy rate of immunodeficient NSG recipients was significantly reduced to 40% (Fig. 3). The average litter size of 4.7 ± 2.9 (Fig.4) was also reduced in comparison with the transplantation of fresh half ovaries into histocompatible recipients. Nevertheless, the 4 fertile recipients produced 12 litters and 50 offspring all together.

### Experiment 3 (cryopreservation)

Results of mating after transplantation of ovarian tissue cryopreserved with 4 different protocols are summarized in Table 1. Observed pregnancies indicate that all used freezing protocols are able to preserve functionality of the frozen-thawed ovarian tissue. Although differences in pregnancy rate and latency between protocol groups are statistically not significant (*p* ≥ 0.05), recipients of group III got on average fastest gravid and produced the highest number of pups per litter (5/6/6/9/0). The litter size of group III was comparable with the control group with fresh grafts (2/6/9/9/0) and significantly higher compared with group I (3/1/1/2/2, *p* ≤ 0.05).

**Table 1:** Reproductive results after transplantation of frozen-thawed ovary halves (a:b = *p* ≤ 0.05; one-way ANOVA)

Group	Pregnant (pos/used)	Latency (days, mean ± SE)	Litter size (mean ± SD)
I	5/5	49.8 ± 4.3	1.8 ± 0.8 ^a^
II	2/5	53.0 ± 2.7	4.0 ± 0.0
III	4/5	34.5 ± 3.8	6.5 ± 1.7 ^b^
VI	4/5	48.5 ± 3.3	3.8 ± 1.7
V (control)	4/5	60.8 ± 4.9	6.3 ± 3.4 ^b^

## Discussion

### Experiment 1

Transplantation of mouse ovarian tissue is a feasible method to rescue strains with insufficient fitness or to preserve female germ cell tissue of mouse models for archiving and distribution to other labs. Practical applications of the method have been published in which ovaries were transferred as whole, half, or quarter pieces. The division of ovarian tissue to different numbers of recipients could influence the possible number of offspring per ovary donor. Unfortunately, a direct comparison of the published results is impossible due to different experimental conditions. Therefore, we conducted a systematic investigation of the reproductive performance of recipients after transfer of half, quarter, and eighth ovary pieces.

Our results confirm that halves and quarters of ovaries as transplant can be successfully used to reconstitute fertility of recipients [2, 6, 12]. Furthermore, we were able to show for the first time that transplantation of eights can also be successfully done. Pregnancy rates (90–100%, Fig. 3) are comparable between the three groups with different sized ovary pieces. Independent of the size of the transplant, mice gave birth in a time range of 21 to 126 days after mating, suggesting no impact of the graft size on latency. However, there was a significant impact of graft size on average litter size, which was reduced for recipients with quarters and eights in comparison with halves (Fig. 4).

Despite the reduction of litter size, a distribution of ovarian tissue samples to 4 or more recipients, instead of only 2, will reduce the impact of technical failure (loss of large transplants due to insufficient wrapping by the bursa) and increase the absolute number of offspring per ovary donor. This may be crucial if donor tissue is severely limited. Two recipients of ovary halves from one donor produced on average about 38 offspring during the mating period of 4 months. In the same time, 4 recipients of quarters gave birth to an average of 54 pups and 8 recipients of eighths to an average of 82 pups.

Nevertheless, we observed a decrease of produced litters and of the average litter size for recipients of eights over the 4-month mating period. The differences were not significant in comparison with the groups of halves and quarters. However, the decrease could indicate the burn out of oocytes after ovary transplantation caused by ischemia and hypoxia [22, 43–48], resulting in an earlier exhaustion of the oocyte pool in smaller ovary pieces.

It is generally assumed that a bilateral ovariectomy will remove all ovarian follicles of the treated female and mating after this procedure results in no pregnancies [8, 11, 12]. Interestingly, however, about 4% of pups in experiment 1 (17/407) and 8% of pups in experiment 2 (4/50) originated from the natural recipient ovary, detected by the presence (experiment 1) or absence (experiment 2) of red fluorescence protein. We assume that after ovariectomy, some follicles remain in the junction between ovary and ovarian hilus where they can develop and ovulate under appropriate hormonal influence (Fig. 6). Sun et al. [49] confirmed this finding but postulated that the remaining follicles have to migrate back into ovarian tissue to mature and ovulate. They did not find developing oocytes in the tissue of the hilus and reported that ovariectomized females without ovary transplantation did not get pregnant. Nevertheless, genotyping of the offspring after ovary transplantation is recommended to exclude unwanted genotypes from further use.

### Experiment 2

Since tissue transplantation requires histocompatibility between donor and recipient, we studied the feasibility of using immunodeficient NSG females as universal recipients for ovarian tissue from mice with rare or undefined genetic background. Transplantation of ovary halves resulted in a significantly lower pregnancy rate and litter size compared with the group with the same graft size of immunocompetent recipients (40% vs. 100% and 4.7 vs. 6.5, respectively). The genetic background of donors was C57BL/6 in both cases and both recipients were inbred. The low pregnancy rate may be attributed to the lower reproductive performance of the immunodeficient strain. Nevertheless, 50 pups were born from only 4 fertile recipients.

Immunodeficient mice are frequently used as recipients for xenotransplantation [15–32]. Reports about transplantation of mouse ovaries into immunodeficient mice are rare. Wang et al. [14] transplanted cryopreserved ovaries heterotopically into the kidney capsule of SCID mice and confirmed functionality of the grafts. In a study with athymic nude mice, ovaries were transplanted orthotopically and the recipients mated afterwards [12]. The result was one pregnant mouse out of four recipients delivering two pups. Homozygous athymic nude mice are generally not recommended for breeding purposes because of insufficient lactation and bad brood care. The use of NSG females as immunodeficient recipients seems to be an improvement and feasible option to generate offspring from non-histocompatible ovary donors.

### Experiment 3

Ovarian cryopreservation is an alternative method to sperm or embryo freezing to archive valuable mouse strains. Several different protocols have been published using different cryprotectants, controlled freezing or vitrification, and cryo vials or straws [2–9, 11–14, 38]. In order to select an easily applicable freezing method with appropriate results, we tested and compared four different protocols—group I: vitrification in cryo vials [13], group II: solid surface vitrification and storage in cryo vials [14], group III: conventional freezing in straws [14], and group IV: conventional freezing in cryo vials [8]. Although the tested protocols differ significantly in their practical application and outcome, we could confirm usability for all in terms of born offspring after transplantation of frozen-thawed ovary halves. In our experiments, controlled freezing in straws with DMSO as penetrating cryoprotectant (group III) provided the best results regarding pregnancy rate, latency, and litter size; each were comparable with fresh transplanted ovarian tissue. This protocol is also preferable because only one penetrating cryoprotectant is used instead of several: and storage of the straws requires significantly less space compared with cryo vials in the liquid nitrogen tank.

In summary, ovary transfer proves to be a valuable technique for rescue of mouse strains with health or fertility problems and for archiving of valuable strains. Splitting of ovaries into more than half pieces improves the efficient usage of ovarian tissue per donor and reduces the impact of technical failure. NSG mice are a feasible option for ovarian tissue transplantation if no histocompatible recipients are available.

In our experience, controlled freezing in straws using a cryostat with DMSO as cryoprotectant provided the most optimal results in terms of produced offspring after thawing and transplantation.
